# Metabolomics-based profiling for quality assessment and revealing the impact of drying of Turmeric (*Curcuma longa* L.)

**DOI:** 10.1038/s41598-022-13882-y

**Published:** 2022-06-18

**Authors:** Mohamed A. Salem, Riham A. El-Shiekh, Alisdair R. Fernie, Saleh Alseekh, Ahmed Zayed

**Affiliations:** 1 Department of Pharmacognosy and Natural Products, Faculty of Pharmacy, Menoufia University, Gamal Abd El Nasr St., Shibin Elkom, 32511 Menoufia Egypt; 2 Max Planck Institute of Molecular Plant Physiology, Am Mühlenberg 1, 14476 Potsdam-Golm, Germany; 3 Department of Pharmacognosy, Faculty of Pharmacy, Cairo University, El-Kasr El-Aini St., Cairo, 11562 Egypt; 4 Center for Plant Systems Biology and Biotechnology, 4000 Plovdiv, Bulgaria; 5 Department of Pharmacognosy, College of Pharmacy, Elguish Street (Medical Campus), Tanta University, Tanta, 31527 Egypt; 6 Institute of Bioprocess Engineering, Technical University of Kaiserslautern, Gottlieb-Daimler-Straße 49, 67663 Kaiserslautern, Germany

**Keywords:** Mass spectrometry, Metabolomics

## Abstract

Turmeric, the rhizomes of *Curcuma longa* L., is one of the top selling spices, food preservatives, and food colorants. In addition, it exhibits health promoting benefits owing to its unique phytochemical composition. Nevertheless, it is commonly subjected to heat drying, hence, the dried powder is the most used form and can easily be adulterated with allied species. Therefore, our research aimed to profile the phytochemical composition and investigate the impact of drying of turmeric. Extraction and fractionation followed by LC- and GC–MS analysis resulted in the identification of a total of 161 metabolites belonged to various phytochemical classes. Moreover, multivariate data analysis identified curcuminoids, terpecurcumins, and organic acids as potential markers for drying. Based on the applied analytical techniques in combination with chemometrics, these investigations have succeeded to provide good coverage of the metabolome of turmeric in both fresh and dried forms.

## Introduction

Food additives, according to the US Food and Drug Administration (FDA), are ''any substance the intended use of which results or may reasonably be expected to result, either directly or indirectly, in its becoming a component or otherwise affecting the characteristics of any food'', while the European Food Safety Authority considers food additives as ''substances added intentionally to foodstuffs to perform certain technological functions, for example to color, to sweeten or to help preserve foods''^1,2^.

Among the natural and generally recognized as safe (GRAS) food additives is turmeric rhizome (*Curcuma longa* L., Zingiberaceae) which either in its fresh or dried powdered form as a major constituent of curry powder^3–5^. Additionally, it is a principal component in many dishes throughout the world as a spice, food preservative and coloring agent, specifically in India, China, and South East Asia^6^. Moreover, in 2010, turmeric was among the top five selling dietary supplements in the United States^7^.

Turmeric, furthermore, is purported to possess various bioactivities and health promoting effects, including anti-inflammatory, antimicrobial, anti-hyperlipidemic, antioxidant, and anti-tumor activities^8,9^. These effects explain its traditional application in Ayurveda and folk medicine for the treatment of diverse diseases, *i.e.*, gastric, hepatic, and infectious diseases^10^. The numerous bioactivities of turmeric rhizome are attributed mainly to its oleoresin richness of wide spectrum of phytochemicals including curcuminoids (1–6% *w/w*), *i.e.*, curcumin, demethoxycurcumin, bisdemethoxycurcumin and calebin-A, and essential volatile metabolites (3–7%), such as zingiberene, curcumene, aromatic turmerone, *α*-turmerone, *β*-turmerone, furanodiene, bisacurone, germacrone, curdione, cyclocurcumin and *α*-santalene^11,12^. Besides, these targets, carbohydrates, moisture, protein, fat, minerals, and fiber constitute 60–70%, 6–13%, 6–8%, 5–10%, 3–7%, and 2–7% *w/w*, respectively^3^. Nevertheless, the fresh rhizome showed different profiles, regarding volatile compounds as revealed by GC/MS. While *α*- and *β*-tumerone are abundant in fresh rhizome, they are minor or less abundant in dry rhizome^11^. Such changes affect the organoleptic properties of the herb, *i.e.*, the fresh form possesses an aromatic and spicy fragrance, yet a distinctive medicinal aroma results upon drying^13^. It is also noteworthy to find that previous reports investigated the effect of various drying methods with focusing on their optimization related to product quality. For instance, Hirun, et al*.* investigated the microwave-vacuum drying approach in terms of microwave power (2400–4000 W) and drying times (10–30 min), where they showed significant effects on the end product’s colour via inhibition of polyphenol oxidase, moisture content, ash, phenolic content, curcuminoid content, and antioxidant activity as well, especially at higher power and longer drying times^5^. Asides, the effect of mechanical drying air employing tray drier was studied in terms of drying air temperatures (45–65 °C) and velocities (1–3 m/sec) on the powder quality^14^. Recently, hot-air dryer coupled with a simulated solar radiation was applied for turmeric drying. The results showed that the dried products possessed intensive orange color. Additionally, curcumin, demethoxycurcumin, and total curcuminoids were influenced, where the lowest curcumin content was detected at 40 °C under PMMA^15^.

Adulteration of turmeric powder, in contrast with the whole fresh or dried rhizome, has been frequently reported including the mixing with *C. zedoaria* (white turmeric) and azo compounds, *i.e.*, metanil yellow and Sudan Red G^4,7^. Detection of these potential contaminants and powder authentication were based on curcumin and dye determination using high-performance liquid chromatography (HPLC), high performance capillary electrophoresis, HPLC-electrospray ionization tandem mass spectrometry (HPLC/ESI–MS), UV–vis spectroscopy coupled with multivariate analysis, and recently with Fourier Transfer-Infrared (FT-IR) and FT-Raman spectroscopic systems^7,16^. In addition, a DNA barcoding method was developed for the detection of plant-based adulterants in turmeric powder^17^. Besides these approaches, the concept of using chemometrics tools, *i.e.*, multivariate data analysis including principal component analysis (PCA), has recently shown to assess quality, discrimination, and authenticate herbal spices based on volatiles, polyphenolics, and curcuminoids profiles^18^.

Drying could be achieved by different methods for example freeze drying, sun drying, microwave drying, oven-drying, among others^19,20^. Various enzymatic and non-enzymatic reactions occur during the drying process which lead to great variation in the bioactivity and chemical composition of the primary and specialized metabolites^21,22^. Metabolomics allows the high-throughput analysis of all metabolites, which is valuable in detection of the possible biotransformation between the primary and secondary metabolites during the food processing^19,21,23^. Changes in the chemical compositions due to the processing methods will lead to several changes in the biological activity of the plant, especially if the beneficial effects are not attributed to one single component, but due to several the constituents found in the plants^20,24–28^. Consequently, the proper method should be designated to optimize the yield of the target metabolites.

Generally, amino acids in their free form are known as the major taste-active compounds in the foodstuffs. The free amino acid compositions were reported to be altered by the different drying process. Additionally, the drying at high temperatures affected the nutrient content, color and physico-chemical properties, sensory characteristics, including aroma, flavor, and texture. Potentially decreased the quality and market value of the functional ingredients by the food industry.

Given the importance of turmeric and its richness in phytochemical composition, a comprehensive analysis of turmeric rhizome is critically required to ensure both safety and quality. In addition, assessment of the impact of drying on its chemical profile is necessary. Therefore, the current research comprehensively characterized the primary and specialized metabolites of fresh and dried turmeric via various modern sensitive chromatographic techniques determining the appropriate form either for nutrition or medicine. In addition, chemometrics tools were applied for the identification of potential markers that can be used for authentication and discrimination between fresh and dried samples.

## Results and discussion

**Figure 1:**
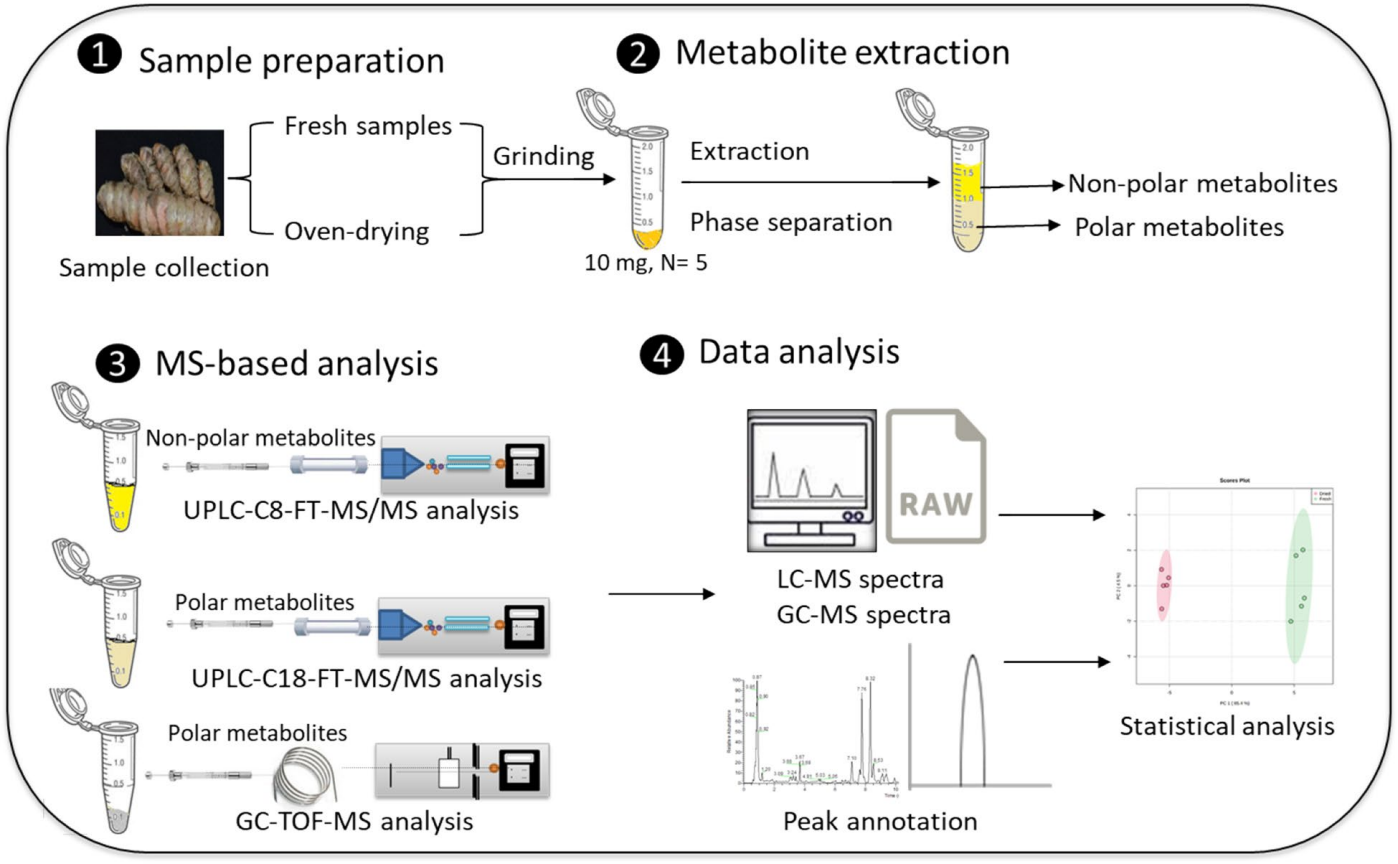
A schematic diagram summarizes the sample preparation, extraction and MS-based analysis of fresh and dried turmeric rhizomes.

In order to cover the diverse and wide spectrum of metabolites in either fresh or dried turmeric rhizomes, a comprehensive extraction protocol was conducted. In addition, various hyphenated analytical methods were performed to identify potential markers in the complex matrix of metabolites and to investigate the effect of drying (Fig. 1).

The application of the recently developed extraction and fractionation protocol allowed a relatively comprehensive profiling of turmeric metabolomes with regards to primary and secondary metabolites. A total of 161 compounds have been annotated (Table S1). They belonged to different chemical classes, including curcuminoids (16), lipids (52), sesquiterpenoids (11), terpecurcumins (10), amino acids (25), sugars (12), organic acids (15), flavonoids and iridoids (2 each), and other miscellaneous compounds (16).

**Figure 2:**
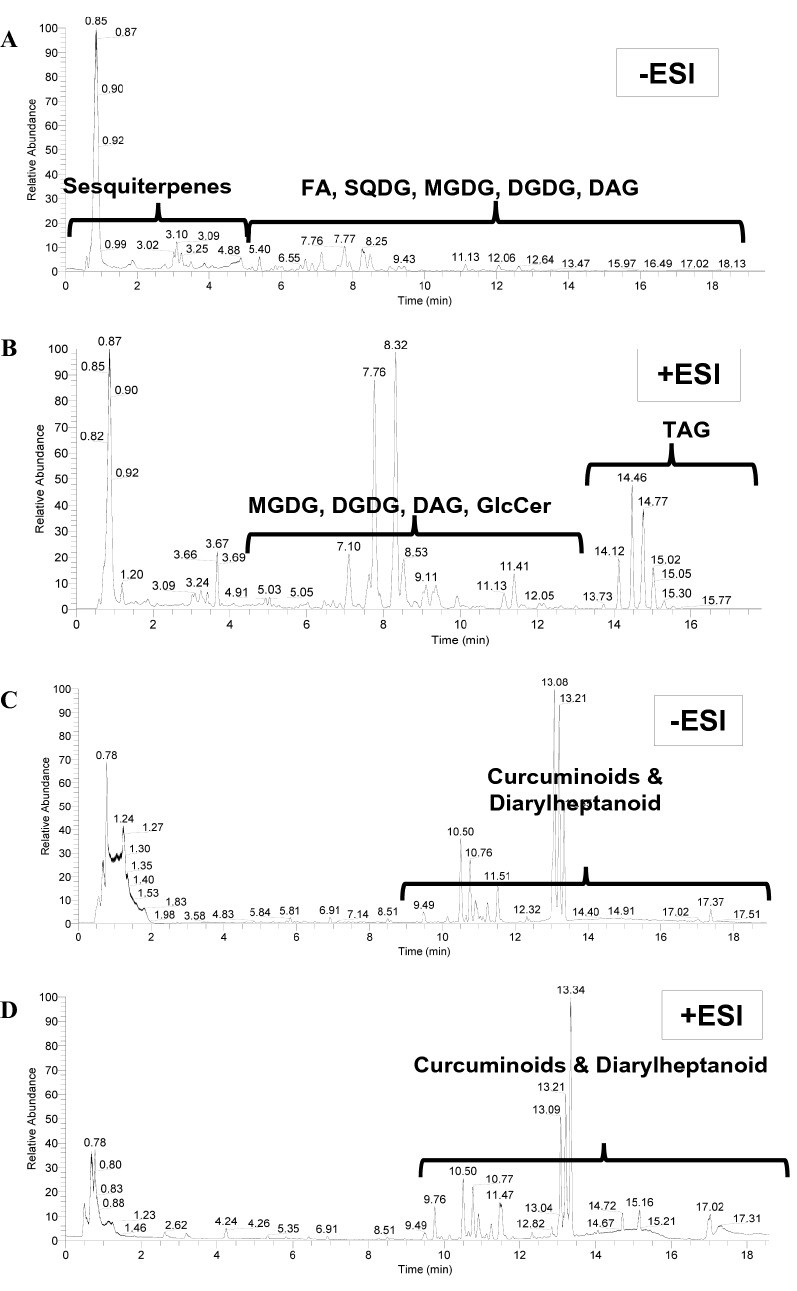
Total ion chromatograms of turmeric rhizome (*Curcuma longa* L.) as analyzed by UPLC-C_8_-FT-MS/MS in negative (**A**) and positive (**B**) ionization modes, and UPLC-C_18_-FT-MS/MS in negative (**C**) and positive (**D**) ionization modes. Mass spectra were recorded in relative abundances of eluted peaks (y-axis) versus retention time in minutes (x-axis). The region of each eluted compounds class is indicated according to its elution window either in positive or negative ionization modes. DAG: diacylglyceride, DGDG: digalactosyldiacylglycerol, ESI: electrospray ionization, FA: fatty acid, GlcCer: glucosylceramide, MGDG: monogalactosyldiacylglycerol, SQDG: sulfoquinovosyldiacylglycerol, TAG: triacylglyceride.

The total ion chromatograms (TIC) of turmeric are shown in Fig. 2A–D as analyzed by UPLC-C_8_-FT-MS/MS and UPLC-C_18_-FT-MS/MS in negative (-ESI) and positive (+ ESI) ionization modes. The retention time range of each eluted class was indicated according to its elution window in either positive or negative ionization modes. Sesquiterpenes were eluted firstly followed by fatty acids (FA), sulfoquinovosyl-diacylglycerol (SQDG), monogalactosyldiacylglycerol (MGDG), digalactosyldiglyceride (DGDG), and diacylglyceride (DAG) in the negative mode, while the MGDG, DGDG, DAG, and glucosylceramide (GlcCer) appeared in front of the triacylglyceride (TAG) of the positive mode of UPLC-C_8_-FT-MS/MS of the non-polar fraction (MTBE fraction layer), Fig. 2A–B. On the other hand, curcuminoids and diarylheptanoidin appeared late starting from the 9th min in either negative or positive mode of UPLC-C_18_-FT-MS/MS of the polar fraction (MeOH/H_2_O layer), as shown in Fig. 2C, D. Each class of metabolites will be covered in the following subsections.

### Curcuminoids

Curcuminoids are recognized as the major metabolites in turmeric rhizomes and are purported to possess a wide range of pharmacological activities such as anti-inflammatory, antioxidant, and cytotoxic activities^29^. Curcuminoids were among the most abundant including their three major forms; bisdemethoxycurcumin, curcumin and demethoxycurcumin.

**Figure 3:**
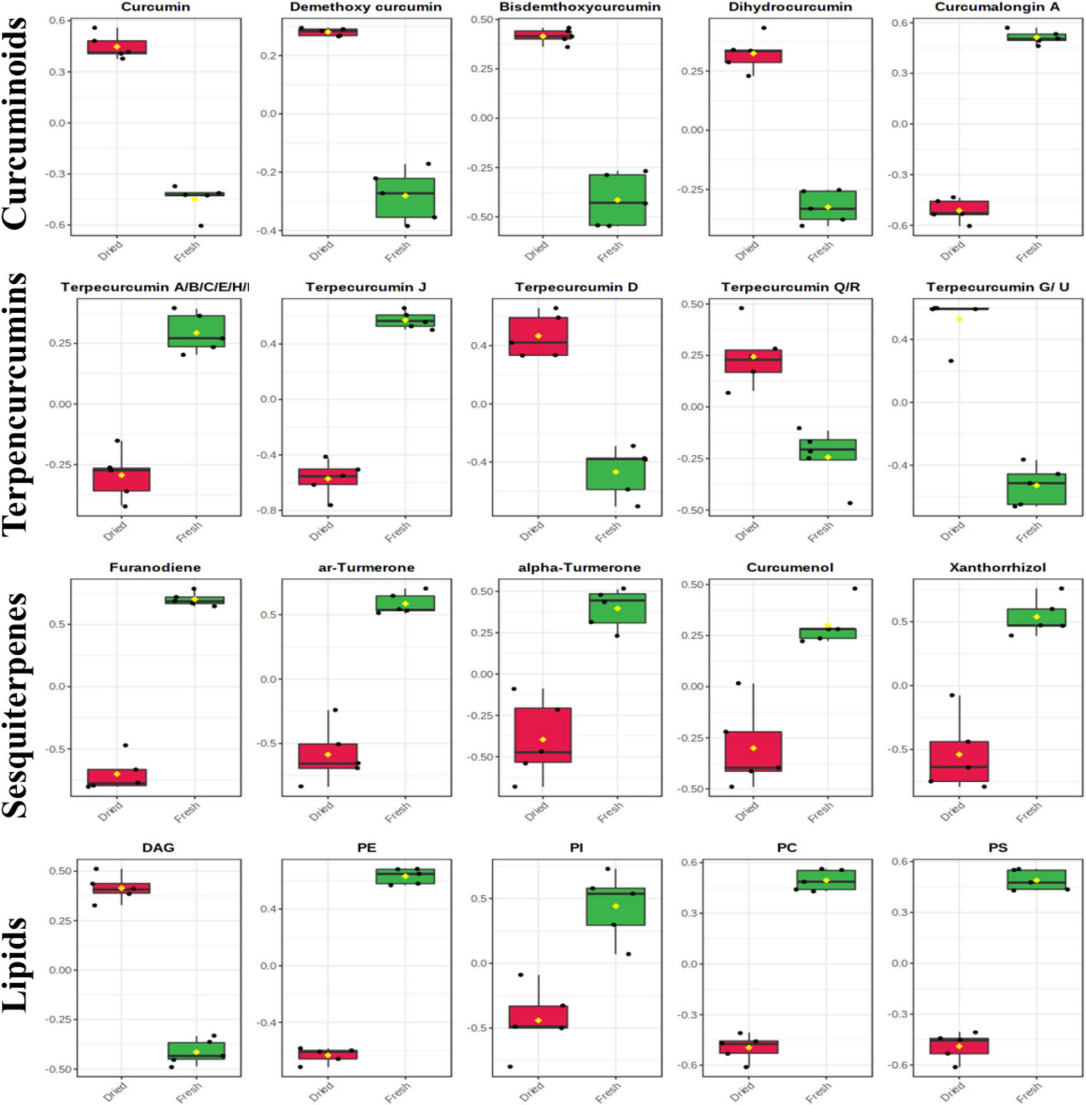
Comparison between the relative abundances of some representative examples of identified metabolites of turmeric (*Curcuma longa* L.) in fresh and dried samples (n = 5). For instance, the figure obviously demonstrates that curcuminoids were more enriched in the dried samples than fresh analogues.

About 16 curcuminoids were identified in the current research in the polar fraction (MeOH/H_2_O layer) of turmeric extract and analyzed by UPLC-C_18_-FT-MS/MS, Table S1. Their relative abundances were affected by drying, where the results demonstrated that curcuminoids abundance increased after herbal drying including all members, in accordance with previous reports^29^, except curcumalongin A and curcumin dimer (Table S1 and Fig. 3). In addition, demethoxy curcumin and bisdemthoxycurcumin were the most abundant metabolites among all members of curcuminoids.

Structural annotation of curcuminoids (curcumin, desmethoxycurcumin, bisdesmethoxycurcumin) (Suppl. Fig. S1) was based on the mass fragments at *m/z* 369, 339, and 309 in positive ion mode and 367, 337, and 307 in negative ion mode with the product ions at *m/z* 217 [M-C_9_H_10_O_2_]^−^ for curcumin, *m/z* 217 [M-C_8_H_8_O]^−^ and 187 [M-C_9_H_10_O_2_]^−^ for desmethoxycurcumin, and *m/z* 187 [M-C_8_H_8_O]^−^ for bisdesmethoxy curcumin, respectively^30^.

### Sesquiterpenoids

Eleven sesquiterpenoids metabolites were detected (Table S1). They were detected mostly in the non-polar MTBE fraction in addition to few numbers in polar MeOH/H_2_O analyzed by UPLC-C_8_-FT-MS/MS and UPLC-C_18_-FT-MS/MS, respectively. The relative abundances of sesquiterpenoids showed clear differences between fresh and dried samples, where all of them were more abundant in fresh than dried samples and likely to be affected by drying process. For instance, the relative abundances of ar-turmerone and furanodiene were decreased approximately to half upon drying, Table S1 and Fig. 3.

Particularly, the bisabolane constituents, *i.e.*, xanthorrhizol followed by *α*-turmerone and ar-turmerone were the most abundant sesquiterpenoids. These results agreed with previous literature confirming that, for example, α-turmerone, and ar-turmerone, are key taxonomic markers for turmeric^31^. However, xanthorrhizol is a unique constituent of *C. xanthorrhiza* Roxb., commonly known as Java turmeric, and a wide variety of biological properties have been reported for this metabolite^29^. Hence, detection of xanthorrhizol as a major constituent of either fresh or dried *C. longa* has been reported for the first time.

### Terpecurcumins

Terpecurcumins are bioactive metabolites isolated from the rhizomes of turmeric. They are synthesized through the hybridization of curcuminoids and bisabolanes^32^. Various terpecurcumins were identified mostly in the non-polar fraction of turmeric and analyzed by UPLC-C_8_-FT-MS/MS. Results revealed that tercurcumin Q and terpecurcumin R were the most abundant members of this class. In addition, their abundances were variable between dry and fresh samples. For example, most of terpecurcumins were more abundant in dried samples, similar to curcuminoids, as terpecurcumin Q, R, and D. However, others as terpecurcumin S, A/B/C, and J were much more in fresh analogues, Table S1 and Fig. 3. The research has thus distinguished between fresh and dried turmeric regarding terpecurcumins for the first time.

### Lipids

Lipids are non-polar primary metabolites that can be classified into different types, including DGDGs, FAs, glucuronosyldiacylglycerols (GlcADGs), MGDG, phosphatidylethanolamines (PEs), phosphatidylserines (PSs), phosphatidylinositols (PIs), sulfoquinovosyl-diacylglycerols (SQDGs), GlcCers, and TAGs. All of them were detected in the non-polar MTBE fraction of turmeric extracts. Mostly, fresh samples were richer in lipid components than the dried counterparts, Table S1 and Fig. 3.

Among the identified lipid components were DGDGs (13), GlcADG (six), MGDG (six), SQDGs (three), TAG (10), and phospholipids (seven). Their abundances were mostly relatively high in fresh samples than dried. Moreover, seven fatty acids were identified including essential unsaturated types as 18:3, 18:1, and 16:1. Their relative abundances were also generally higher in fresh samples. Essential fatty acids are considered key nutrients that affect growth, development and nutrition-related chronic disease^33^.

### Other miscellaneous compounds

Amino acids, sugars, and organic acids were detected in polar fraction following derivatization and analysis by GC–TOF–MS. As shown in Suppl. Fig. S2, pyroglutamate, alanine, serine, asparagine, aspartate, and glycine were the most abundant among amino acids. These metabolites were shown to decrease upon drying.

In addition, the abundances of sugars were essentially similar between fresh and dried samples, except in case of raffinose which highly increased after drying. Sucrose and *myo*-inositol were the most abundant and additionally were relatively more abundant in fresh samples. Such abundant sugars may contribute to the sweet taste of the fresh rhizomes, especially given the lower abundances of curcuminoids in the fresh rhizomes. *myo*-Inositol has purported health promoting effects, where it is suggested to provide promising effects against different diseases including diabetes and cancer^34^. The levels of *myo*-inositol were not significantly affected by drying, indicating that the dried form is also effective. Moreover, the content of the trisaccharide raffinose was nearly double in dried samples indicating that it may be a byproduct of a certain decomposition reaction induced by heating. Interestingly in plants, its presence is always associated with protection against oxidative damage^35^.

Furthermore, the level of organic acids and the effect of drying on their abundances were investigated. Citric acid was the most abundant in both fresh and dried samples. Yet, its abundance was higher in fresh rhizomes. Other major acids were 4-amino-butanoic acid, fumaric acid, and gluconic acid. However, effect of drying on the levels of these compounds was unclear and variable. While 4-amino-butanoic acid, gluconic acid, and 1-dehydro-ascorbic acid were more in fresh samples, others as fumaric acid, glyceric, and pyruvic acids were in dried samples.

Also, *β*-citraurol and *β*-citraurin are among apocarotenoids that are derived from carotenoids through oxidative cleavage, and hence may affect the powder color. The effect of drying was relatively different, *i.e.*, while *β*-citraurol was more abundant in fresh samples, *β*-citraurin was in dried counterparts. Flavonoids were also among the identified compounds in turmeric, which have not been well investigated in previous literature. The current research could identify two flavonoid glycosides, *i.e.*, hesperidin and quercetin di-rhamnoside, in the MeOH/H_2_O fraction. The results showed that they were richer in fresh samples, Suppl. Fig. S2.

Interestingly, the research revealed the presence of compounds in turmeric for the first time. Examples included drovomifoliol-*O*-glucopyranoside, oleuropeoylsucrose, and corchoionoside B which were previously detected in the Egyptian Murcott mandarin waste^36^, myrtaceous species^37^, and Vietnamese *Corchorus olitorius* L.^38^, respectively. The presence of these compounds would increase the medicinal interest and the importance of turmeric.

### Multivariate data analysis of turmeric samples

**Figure 4:**
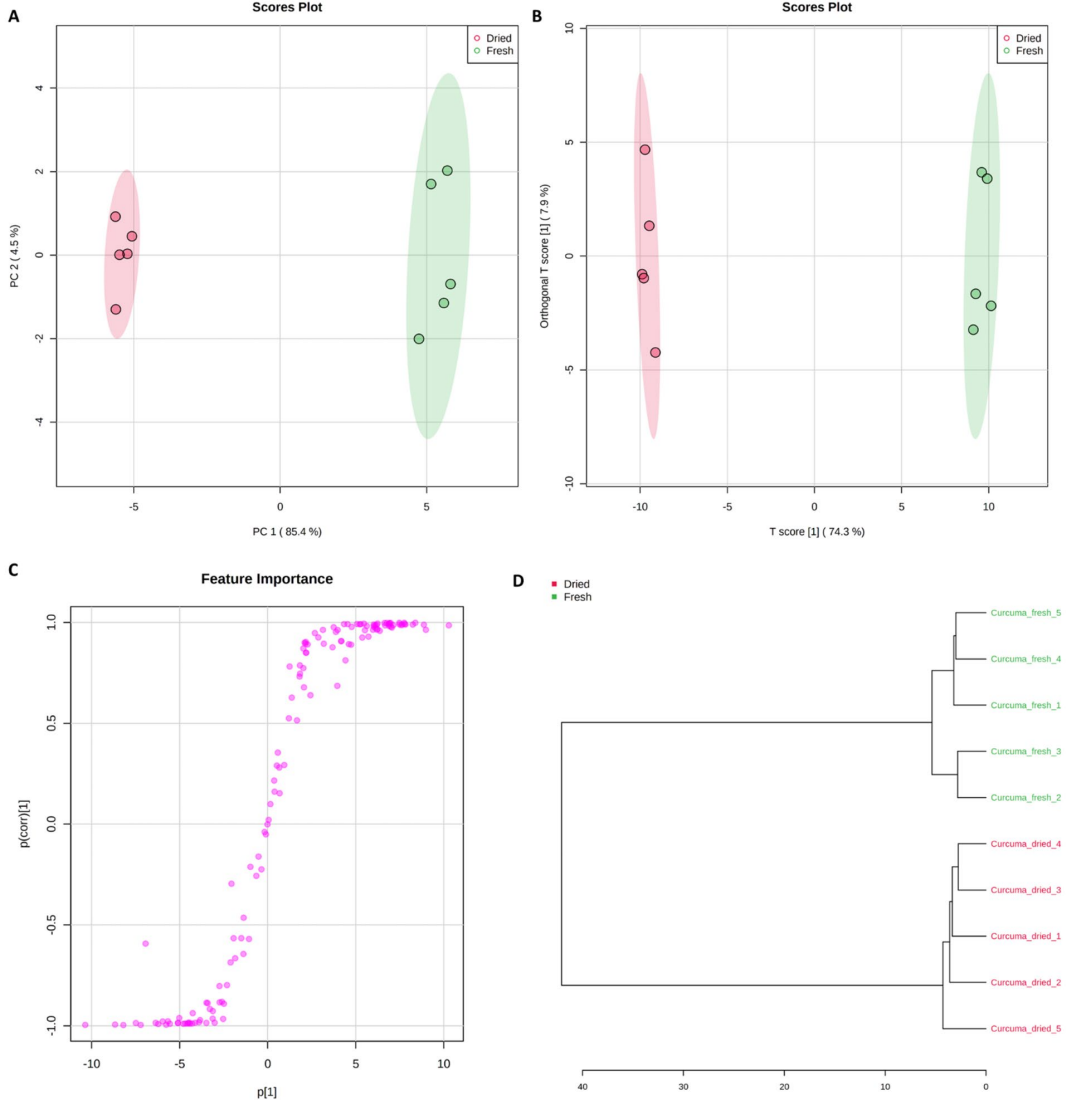
(**A**) Principal Component Analysis (PCA) score plot of fresh and dried turmeric (*Curcuma longa* L.) samples (n = 5). A complete separation is shown between both samples mainly on the PC1 (85.4%); (**B**) Orthogonal Projections to Latent Structures Discriminant Analysis (OPLS-DA) score plot which also confirms the PCA results; (**C**) OPLS-DA S-plot from which raffinose, threose, glycolic, fumaric, pyruvic acids, and monomethyl curcumin potentially marked dried samples, while metabolites such as amino acids and sesquiterpenoids were more associated with fresh rhizomes. (**D**) Hierarchical cluster analysis (HCA) of fresh and dried turmeric samples using Ward´s clustering algorithm.

A principal component analysis (PCA) score plot revealed a clear segregation of the dried from fresh samples of turmeric mainly across PC1 representing 85.4% of the total variance (Fig. 4A). In addition, Orthogonal Projections to Latent Structures Discriminant Analysis (OPLS-DA) score plot confirmed the PCA findings, Fig. 4B. The OPLS-DA S-plot demonstrated that raffinose threose, glycolic, fumaric, pyruvic acids, and monomethyl curcumin potentially marked dried samples, while metabolites such as amino acids and sesquiterpenoids were more associated with fresh rhizomes, Fig. 4C. Hierarchical cluster analysis (HCA) revealed separation of fresh and dry samples to two different clusters reflecting the metabolic composition (Fig. 4D).

**Figure 5:**
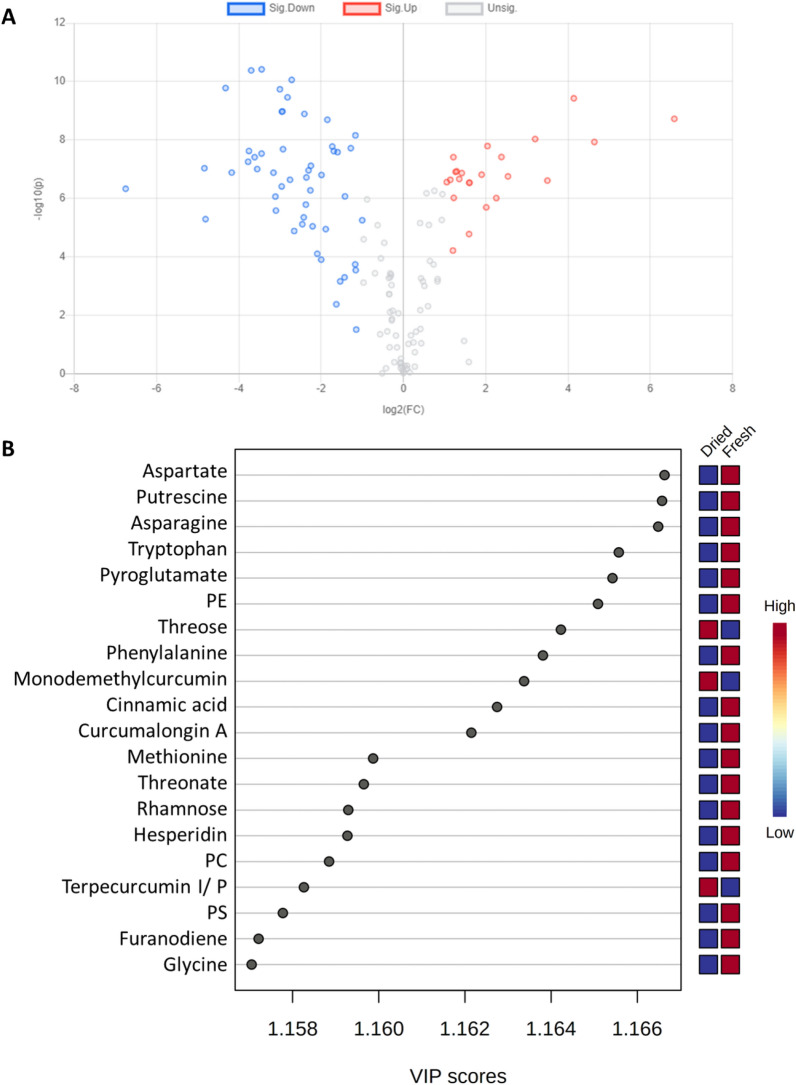
(**A**) Volcano plot showing effect of drying on turmeric rhizomes, where metabolites on the right side (positive side) increased significantly upon drying, while metabolites on the left side (negative side) were more associated with fresh rhizomes. (**B**) VIP scores showing the distribution of potential markers between fresh and dried samples. Colored boxes on right indicate relative abundance of the corresponding metabolite for fresh and dried samples.

In addition, a Volcano plot was traced to show the impact of drying. The results showed that at *p* < 0.05, the relative abundances of 51 metabolites were decreased upon drying (negative side), while 23 increased (positive side) and 87 were not significantly affected. Examples of metabolites that decreased by drying mainly belonged to amino acids such as glutamine, tryptophan, serine and homoserine, as well as glutathione (oxidized form). In contrast, threose, mono demethylcurcumin, and glycolic and fumaric acids were examples of metabolites that were enriched upon drying in turmeric rhizomes, Fig. 5A.

A variable importance in projection (VIP) plot displayed the top 20 most important metabolite features identified by OPLS-DA (Fig. 5B). For instance, amino acids and phospholipids can be recognized as markers for fresh turmeric, while terpecurcumins and tetrahydrodemethoxycurcumin are for dried counterparts. Most of the previously published articles have focused on quantitative and qualitative profiling of curcuminoids and essential oils contents of turmeric oleoresin. However, the current research has succeeded to profile the phytochemical composition of turmeric more comprehensively following application of an efficient metabolomics approach. The presented workflow is a powerful tool for the quality assessment, authentication and discrimination between different forms of food or herbal medicine.

### Conclusion

A relatively comprehensive phytochemical analysis combined with multivariate analyses of food spices attracted considerable attention as a potential quality assessment tool. Since drying is still the most applicable and ancient method for herb and food preservation, the effect of drying has been studied regarding the nutritional, organoleptic characteristics, and medicinal use determining the most suitable form for each use. The impact of heat drying was investigated on turmeric rhizomes within metabolomics-based study for the first time. Intriguingly, most classes of lipids, sesquiterpenoids, flavonoid glycosides, and amino acids were more abundant in fresh samples, while curcuminoids and terpecurcumins increased by drying. Hence, the nutritional value and aromatic property may be decreased by drying, in contrast to the medicinal applications. This point raised the questions of how to optimize the drying process getting both nutritional and full aromatic values of turmeric beside the medicinal benefits.

## Material and methods

### Plant materials

Fresh *Curcuma longa* L. (The Northern region, NR-1, orange variety, organic turmeric from local field, Upala, Costa Rica) rhizomes were provided by BIO-COMPANY® GmbH (Berlin, Germany). No permissions were requested to order the plant samples. The material was authenticated by Eng. Theres Labib, Consultant of Plant Taxonomy at Ministry of Agriculture, Egypt. Voucher specimens (01-10-2019) were kept in the herbarium of Department of Pharmacognosy and Natural Products, Faculty of Pharmacy, Menoufia University, Menoufia, Egypt. All methods were performed in accordance with the relevant guidelines and regulations.

### Preparation and drying of samples

Firstly, the outer cork tissues were removed carefully with a knife. Then, the peeled rhizomes were cut into small pieces (1 mm in length) of the same thickness. In parallel, a portion of the peeled turmeric rhizomes was lab-dried by subjecting to an oven adjusted at 40 °C for 1.5 days^39–41^. The selection of drying time and temperature was based on the preliminary trials for optimum drying without burning or inducing severe changes in the color of the rhizomes as well as the available reported literature^42–44^. Previous studies found that the turmeric rhizomes that were subjected to oven-drying at 60–100 °C showed changes in total phenolic content, colour value as well as polyphenol oxidase activity^43^. The time of drying was based on preliminary tests to reach the final moisture contents below 10% (wet basis). Afterwards, samples (five biological replicates/condition) were labeled and kept frozen in liquid nitrogen until further extraction steps.

### Sample preparation for analysis

#### Extraction protocol and phase separation

Frozen turmeric samples were homogenized and then subjected to our previously developed extraction and fractionation protocol^45^. In brief, 10 mg of each replicates, calculated on dry weight basis, were incubated in a micro-centrifugation tube with pre-cooled (1 mL, −20 °C) methyl *tert*-butyl ether (MTBE):methanol (MeOH) (3:1, *v/v*). Then, a phase-separation step was carried-out with 650 µL H_2_O/MeOH (3:1, *v/v*). This fractionation step resulted in two different layers, including an upper non-polar fraction of MTBE and a polar fraction in the lower MeOH/H_2_O layer.

#### Preparation of samples for MS-based analysis

For analysis of the non-polar fraction, aliquots (500 µL) from the MTBE layer were dried for 3 h in a vacuum centrifuge. The dried pellets were resuspended in 400 µL acetonitrile:isopropanol (70:30) before they were subjected to UPLC-C_8_-FT-MS/MS analysis. Semi-polar to polar metabolites were analyzed via two independent aliquots (300 and 150 µL) from MeOH/H_2_O layer. Both aliquots were dried in a vacuum centrifuge for 6 h. Then, the dried pellets, resulted from the 300 µL aliquots, were resuspended in 200 µL 50% aqueous MeOH before they were subjected to UPLC-C_18_-FT-MS/MS analysis^46^. The other dried pellets, resulted from of 200 µL aliquots, were derivatized before they were subjected to GC–TOF–MS analysis^47^.

#### Chromatography coupled with mass spectrometry instrumentation

The UPLC-FT-MS/MS consisted of a Reversed Phase (RP) column (100 × 2.1 mm, 1.7 μm diameter particles, Waters®) in a Waters Acquity UPLC system (Waters®, Manchester, UK). The chromatography unit was coupled to a mass spectrometer able to work in both ionization modes, *i.e.*, positive and negative, using a heated electrospray ionization (HESI) source together with Orbitrap-type MS (Exactive, Thermo-Fisher®, Bremen, Germany)^47^. Additionally, GC–TOF–MS was performed for derivatized samples with MSTFA^48^.

### Data analysis

The LC or GC datasets were imported into Microsoft excel (Excel 2010, Microsoft®, Redmond, USA). Only significant peaks, *i.e.*, with at least five-fold higher intensity in comparison to the blank peaks, were further considered for data analysis. Metabolite data obtained were then normalized by weight and internal standard normalization. All statistical analyses were performed using the algorithms embedded within Microsoft excel. In addition, multivariate data analysis (MVDA) was conducted using Metaboanalyst 3.0 software^49^.

## Supplementary Information

Supplementary Information.

## Data Availability

All data generated during this study are included in this published article and its supplementary information files.
