# Supplementary material for: Metabolomics-based profiling for quality assessment and revealing the impact of drying of Turmeric (Curcuma longa L.)
Source: Sci Rep. 2022 Jun 18;12:10288. doi: 10.1038/s41598-022-13882-y (PMC9206664; doi:10.1038/s41598-022-13882-y)
Supplement: Supplementary file 1 — Supplementary Information. [file 41598_2022_13882_MOESM1_ESM.docx]

**Supplementary materials**

**Supplementary Figures**

**
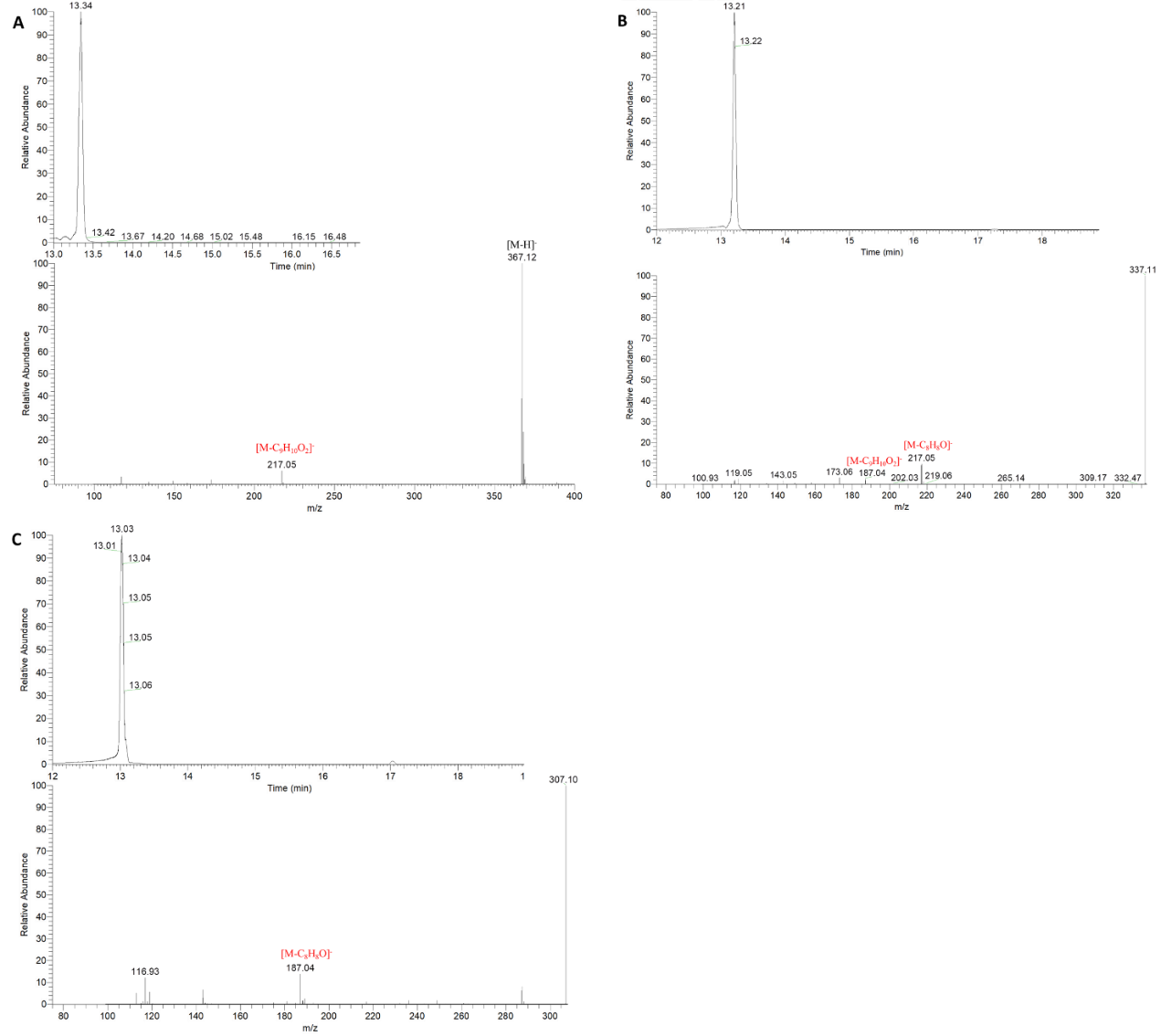
**

**Figure S1:** Identification of curcuminoids (curcumin, desmethoxycurcumin, bisdesmethoxycurcumin)

**(A)** Total ion chromatogram of curcumin in negative ionization modes and mass spectrum at RT 13.34 min. **(B)** Total ion chromatogram of desmethoxycurcumin in negative ionization modes and mass spectrum at RT 13.21 min. **(C)** Total ion chromatogram of bisdesmethoxycurcumin in negative ionization modes and mass spectrum at RT 13.04 min.

| **Flavonoids** | | | | | | |
| --- | --- | --- | --- | --- | --- | --- |
| **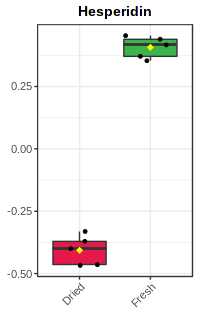** | | **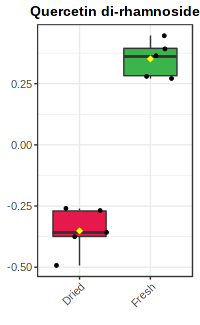** | |  | |  |
| **Amino acids** | | | | | | |
| 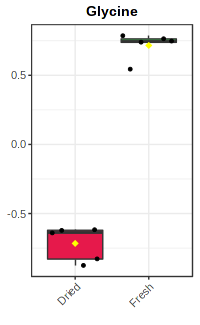 | 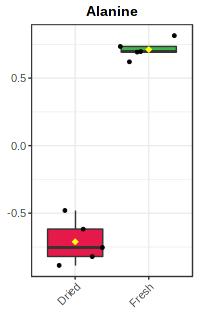 | | 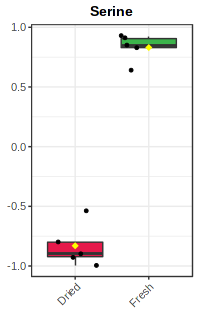 | 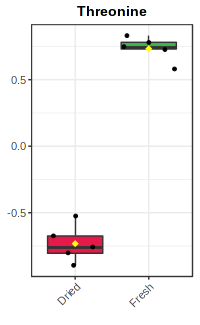 | 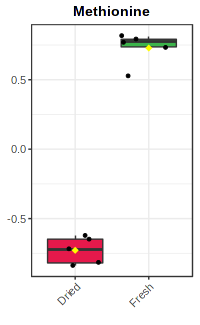 | |
| 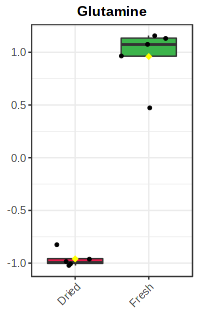 | 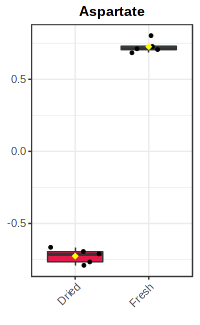 | | 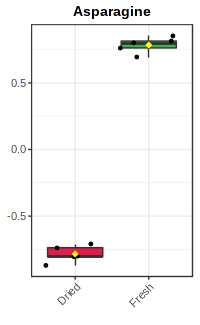 |  | | |
| **Organic acids** | | | | | | |
| 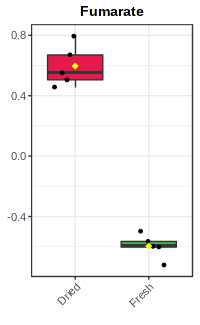 | 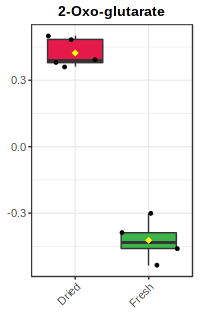 | | 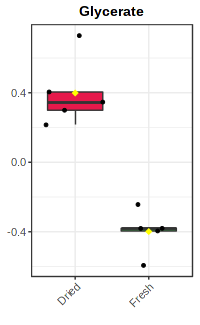 | 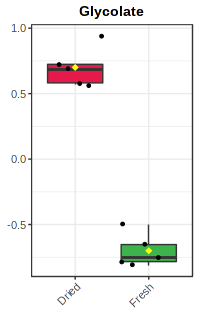 |  | |
| **Others** | | | | | | |
| **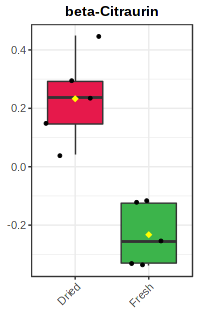** | **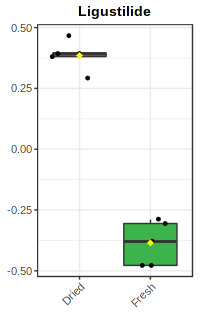** | | **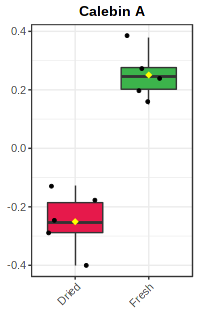** | **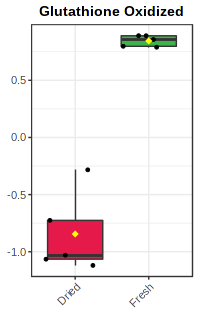** | **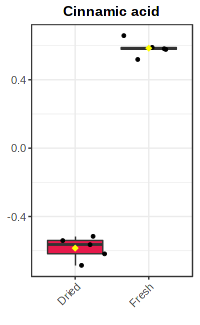** | |

**Suppl. Figure S2**: Comparison between the relative abundances of some metabolites, *i.e.*, amino acids and organic acids, detected in turmeric (*Curcuma longa* L.) in fresh and dried samples. Flavonoids and amino acids were richer in fresh samples, while organic acids in dried analogues.

**Supplementary Tables**

**Table S1:** Relative abundances of tentatively identified metabolites in turmeric fresh and dried rhizomes following application of a comprehensive extraction protocol, fractionation, and analysis by LC-MS/MS and GC-TOF-MS.

| **Metabolite #** | **Phase and technique of detection** | **Compound name** | **Molecular formula** | **Relative abundances ± SD (x10^4^)** | |
| --- | --- | --- | --- | --- | --- |
|  |  |  |  | **Fresh sample** | **Dried samples** |
| **Curcuminoids** | | | | | |
|  | - Polar metabolites in MeOH/H_2_O phase    - UPLC-C_18_-FT-MS/MS | 1,2-dihydrobis(de-*O*-methyl)-curcumin | C_19_H_18_O_4_ | 367.2±43.4 | 922.1±53.0 |
|  |  | Bisdemthoxycurcumin | C_19_H_16_O_4_ | 2198.7±304.3 | 5078±198.1 |
|  |  | Curcumalongin A | C_20_H_16_O_6_ | 2182.7±111.3 | 591.8±50.6 |
|  |  | Curcumalongin B | C_21_H_18_O_7_ | 503.7±79.7 | 1517.1±75.4 |
|  |  | Curcumin | C_21_H_20_O_6_ | 728.6±68.8 | 1887.5±160.7 |
|  |  | Curcumin dimer | C_42_H_38_O_12_ | 25.1±22.2 | 21.0±18.9 |
|  |  | Demethoxy curcumin | C_20_H_18_O_5_ | 3851.7±238.1 | 5670.1±51.8 |
|  |  | Didemethoxybisabolocurcumin ether | C_34_H_38_O_6_ | 12.8±1.5 | 17.6±1.8 |
|  |  | Dihydrocurcumin | C_21_H_22_O_6_ | 644.8±35.4 | 1062.3±65.7 |
|  |  | Dihydrodemethoxycurcumin | C_20_H_20_O_5_ | 113.2±8.3 | 242.6±20.7 |
|  |  | *keto*-curcumin | C_21_H_18_O_7_ | 503.7±79.7 | 1517.1±75.4 |
|  |  | Monodemethylcurcumin | C_20_H_18_O_6_ | 2.3±0.6 | 215.2±29.9 |
|  |  | Tetrahydrodemethoxycurcumin | C_20_H_24_O_5_ | 2.2±0.1 | 2.9±0.1 |
|  |  | Tetrahydrodimethoxycurcumin | C_19_H_20_O_4_ | 2.2±0.1 | 4.0±0.4 |
|  |  | *Bis*(3-4-dihydroxybenzylidene) cyclopentanone | C_19_H_16_O_5_ | 214.7±18.7 | 26.8±3.3 |
|  |  | 1-7-*Bis*(4-hydroxyphenyl)-3-5-heptanediol | C_21_H_20_O_6_ | 0.3±0.3 | 1.8±0.0 |
| **Lipids** | | | | | |
| **Digalactosyldiacylglycerol (DGDG)** | | | | | |
|  | - Non -polar metabolites in MTBE phase  - UPLC-C_8_-FT-MS/MS | DGDG (34:1) | C_49_H_90_O_15_ | 2.2±0.2 | 1.8±0.1 |
|  |  | DGDG (34:2) | C_49_H_88_O_15_ | 11.5±1.1 | 9.63±0.7 |
|  |  | DGDG (34:3) | C_49_H_86_O_15_ | 3.0±0.2 | 2.2±0.2 |
|  |  | DGDG (35:2) | C_50_H_90_O_15_ | 0.2±0.0 | 0.2±0.0 |
|  |  | DGDG (36:1) | C_51_H_94_O_15_ | 0.3±0.0 | 0.3±0.0 |
|  |  | DGDG (36:2) | C_51_H_92_O_15_ | 2.3±0.1 | 1.8±0.2 |
|  |  | DGDG (36:3) | C_51_H_90_O_15_ | 1.1±0.1 | 1.3±0.1 |
|  |  | DGDG (36:4) | C_51_H_88_O_15_ | 3.9±0.3 | 3.2±0.2 |
|  |  | DGDG (36:5) | C_51_H_86_O_15_ | 3.1±0.2 | 2.2±0.2 |
|  |  | DGDG (36:6) | C_53_H_88_O_17_ | 4.7±0.3 | 3.1±0.3 |
|  |  | DGDG (38:2) | C_53_H_96_O_15_ | 0.2±0.0 | 0.2±0.0 |
|  |  | DGDG (40:2) | C_55_H_100_O_15_ | 0.2±0.0 | 0.1±0.0 |
|  |  | DGDG (42:2) | C_57_H_104_O_15_ | 9.0±0.6 | 7.3±0.6 |
| **Fatty acids (FA)** | | | | | |
|  | - Polar metabolites in MeOH/H_2_O phase  - GC-TOF-MS | FA (9:0) | C_9_H_18_O_2_ | 0.4±0.1 | 0.3±0.1 |
|  |  | FA (16:0) | C_16_H_32_O_2_ | 0.7±0.1 | 0.8±0.2 |
|  |  | FA (17:0) | C_17_H_34_O_2_ | 0.1±0.1 | 0.4±0.1 |
|  | - Non -polar metabolites in MTBE phase  - UPLC-C_8_-FT-MS/MS | FA (16:1) | C_16_H_30_O_2_ | 0.4±0.1 | ND |
|  |  | FA (18:1) | C_18_H_34_O_2_ | 0.3±0.1 | 0.3±0.1 |
|  | - Polar metabolites in MeOH/H_2_O phase  - GC-TOF-MS | FA (18:3) | C_18_H_30_O_2_ | 0.3±0.0 | 0.5±0.0 |
|  |  | FA (26:0) | C_26_H_52_O_2_ | 0.1±0.1 | 0.1±0.1 |
| **Glucoronosyldiacylglycerol (GlcADG)** | | | | | |
|  | - Non -polar metabolites in MTBE phase  - UPLC-C_8_-FT-MS/MS | GlcADG (36:0) | C_45_H_84_O_11_ | 0.8±0.1 | 0.7±0.1 |
|  |  | GlcADG (36:1) | C_45_H_82_O_11_ | 3.9±0.3 | 3.5±0.2 |
|  |  | GlcADG (36:4) | C_45_H_76_O_11_ | 0.7±0.0 | 0.4±0.1 |
|  |  | GlcADG (38:1) | C_47_H_86_O_11_ | 0.3±0.0 | 0.3±0.0 |
|  |  | GlcADG (38:3) | C_47_H_82_O_11_ | 0.7±0.1 | 0.7±0.1 |
|  |  | GlcADG (40:9) | C_49_H_74_O_11_ | 3.1±0.2 | 5.5±0.4 |
| **Monogalactosyldiacylglycerol (MGDG)** | | | | | |
|  | - Non -polar metabolites in MTBE phase  - UPLC-C_8_-FT-MS/MS | MGDG (34:3) | C_43_H_78_O_10_ | 0.4±0.2 | 0.4±0.1 |
|  |  | MGDG (36:2) | C_45_H_82_O_10_ | 0.3±0.3 | 0.1±0.0 |
|  |  | MGDG (36:5) | C_45_H_76_O_10_ | 2.8±0.2 | 2.4±0.2 |
|  |  | MGDG (38:2) | C_47_H_86_O_10_ | 0.7±0.0 | 0.6±0.0 |
|  |  | MGDG (43:5) | C_52_H_90_O_10_ | 0.8±0.1 | 0.5±0.1 |
|  |  | MGDG (52:5) | C_61_H_108_O_10_ | 0.2±0.0 | 0.7±0.1 |
| **Phospholipids** | | | | | |
|  | - Non -polar metabolites in MTBE phase  - UPLC-C_8_-FT-MS/MS | PE (28:0) | C_33_H_65_O_8_NP | 1.7±0.2 | 26.6±1.5 |
|  |  | PE (30:0) | C_35_H_69_O_8_NP | 0.7±0.1 | 0.7±0.1 |
|  |  | PE (32:0) | C_39_H_74_O_8_NP | 303.2±25.3 | 7.4±0.7 |
|  |  | PE (36:4) | C_41_H_74_O_8_NP | 34.0±1.9 | 33.8±3.3 |
|  |  | PE (36:2) | C_41_H_78_O_8_NP | 12.9±1.3 | 0.3±0.0 |
|  |  | PI (33:0) | C_42_H_80_O_13_P | 0.2±0.0 | ND |
|  |  | PS (40:6) | C_46_H_77_O_10_NP | 12.1±4.1 | 4.6±1.6 |
| **Sulfoquinovosyl-diacylglycerol (SQDG)** | | | | | |
|  | - Non -polar metabolites in MTBE phase  - UPLC-C_8_-FT-MS/MS | SQDG (38:5) | C_47_H_80_O_12_ | 29.5±1.9 | 25.9±2.5 |
|  |  | SQDG (38:6) | C_47_H_78_O_12_ | 25.5±2.7 | 19.2±1.4 |
|  |  | SQDG (50:4) | C_59_H_106_O_12_ | 3.5±0.3 | 2.8±0.2 |
| **Triacylglycerols (TAG)** | | | | | |
|  | - Non -polar metabolites in MTBE phase  - UPLC-C_8_-FT-MS/MS | TAG (50:3) | C_53_H_96_O_6_ | 0.2±0.0 | 0.2±0.0 |
|  |  | TAG (52:4) | C_55_H_98_O_6_ | 2.0±0.2 | 1.9±0.1 |
|  |  | TAG (52:5) | C_55_H_96_O_6_ | 4.8±0.7 | 4.3±0.4 |
|  |  | TAG (52:6) | C_55_H_94_O_6_ | 0.8±0.1 | 0.8±0.1 |
|  |  | TAG (54:4) | C_57_H_102_O_6_ | 0.3±0.0 | 0.3±0.0 |
|  |  | TAG (54:6) | C_57_H_98_O_6_ | 5.2±0.3 | 5.0±0.3 |
|  |  | TAG (54:7) | C_57_H_96_O_6_ | 9.0±0.6 | 8.5±0.5 |
|  |  | TAG (54:8) | C_57_H_94_O_6_ | 4.2±0.4 | 3.9±0.2 |
|  |  | TAG (54:9) | C_57_H_92_O_6_ | 0.4±0.0 | 0.3±0.0 |
|  |  | TAG (56:5) | C_59_H_10_4O_6_ | 0.1±0.0 | 0.1±0.0 |
| **Sesquiterpenoids** | | | | | |
|  | - Non -polar metabolites in MTBE phase  - UPLC-C_8_-FT-MS/MS | Curcumenolactone A/B | C_15_H_20_O_3_ | 2.9±0.3 | 2.3±0.2 |
|  |  | Zederol | C_15_H_20_O_3_ | 2.8±0.3 | 2.3±0.2 |
|  |  | ar-Turmerone | C_15_H_20_O | 113.5±13.3 | 65.5±9.2 |
|  |  | Zedoarondiol | C_15_H_24_O_3_ | 0.8±0.1 | 0.2±0.0 |
|  |  | Turmeronol A/B | C_15_H_20_O_2_ | 17.1±1.1 | 6.4±1.0 |
|  |  | Curzerenone | C_15_H_18_O_2_ | 5.1±0.8 | 1.1±0.2 |
|  |  | Furanodiene | C_15_H_20_O | 46.2±4.5 | 4.2±1.1 |
|  |  | Zederone | C_15_H_18_O_3_ | 13.8±1.0 | 9.5±0.9 |
|  | - Polar metabolites in MeOH/H_2_O phase    - UPLC-C_18_-FT-MS/MS | Curcumenol | C_15_H_24_O_2_ | 8.5±0.7 | 5.2±0.9 |
|  |  | α-Turmerone | C_15_H_24_O | 209.0±25.7 | 92.8±23.9 |
|  |  | Xanthorrhizol | C_15_H_22_O | 2033.7±434.8 | 476.6±221.4 |
| **Terpecurcumins** | | | | | |
|  | - Non -polar metabolites in MTBE phase  - UPLC-C_8_-FT-MS/MS | Terpecurcumin V/W | C_31_H_36_O_7_ | 1.0±0.1 | 0.8±0.1 |
|  |  | Terpecurcumin J | C_34_H_40_O_6_ | 2.7±0.2 | 0.6±0.1 |
|  |  | Terpecurcumin Q/R | C_36_H_42_O_6_ | 25.1±2.3 | 34.8±3.5 |
|  |  | Terpecurcumin S | C_34_H_40_O_5_ | 10.1±0.6 | 5.0±0.7 |
|  |  | Terpecurcumin L/M/N/O/T | C_36_H_44_O_6_ | 3.9±0.3 | 3.8±0.2 |
|  |  | Terpecurcumin A/B/C/E/H/K | C_36_H_44_O_7_ | 8.0±0.5 | 5.2±0.4 |
|  |  | Terpecurcumin F | C_36_H_62_O_8_ | 3.2±0.2 | 2.6±0.2 |
|  |  | Terpecurcumin D | C_36_H_42_O_8_ | 4.4±0.9 | 13.3±2.4 |
|  |  | Terpecurcumin G/U | C_35_H_40_O_7_ | 1.9±0.3 | 7.8±1.3 |
|  |  | Terpecurcumin I/P | C_36_H_42_O_7_ | 2.1±0.3 | 10.9.4±1.0 |
| **Flavonoids** | | | | | |
|  | - Polar metabolites in MeOH/H_2_O phase  - UPLC-C_18_-FT-MS/MS | Hesperidin | C_28_H_34_O_15_ | 13.7±0.6 | 6.1±0.4 |
|  |  | Quercetin di-rhamnoside | C_27_H_30_O_15_ | 1.2±0.1 | 0.6±0.1 |
| **Iridoids** | | | | | |
|  | - Polar metabolites in MeOH/H_2_O phase    - UPLC-C_18_-FT-MS/MS | Morroniside (iridoid glycoside) | C_17_H_26_O_11_ | 0.3±0.0 | 0.1±0.0 |
|  |  | Secologanin | C_17_H_24_O_10_ | 52.3±1.8 | 57.0±5.2 |
| **Amino acids** | | | | | |
|  | - Polar metabolites in MeOH/H_2_O phase  - GC-TOF-MS | Alanine | C_3_H_7_NO_2_ | 97.2±19.2 | 7.6±1.4 |
|  |  | Asparagine | C_4_H_8_N_2_O_3_ | 30.7±8.0 | 1.5±0.4 |
|  |  | Aspartic acid | C_4_H_7_NO_4_ | 66.4±10.8 | 4.9±1.3 |
|  |  | Glutamine | C_5_H_10_N_2_O_3_ | 2.1±1.0 | 0.02±0.0 |
|  |  | Glycine | C₂H₅NO₂ | 17.1 ±4.4 | 1.3±0.2 |
|  |  | Histidine | C_6_H_9_N_3_O_2_ | 0.3±0.2 | 0.1±0.0 |
|  |  | Homoserine | C_4_H_9_NO_3_ | 0.1±0.0 | 0.0±0.0 |
|  |  | Isoleucine | C_6_H_13_NO_2_ | 1.6±0.4 | 0.2±0.0 |
|  |  | Leucine | C_6_H_13_NO_2_ | 1.5±0.5 | 0.3±0.1 |
|  |  | Methionine | C_5_H_11_NO_2_S | 0.6±0.2 | 0.0±0.0 |
|  |  | Methionine sulfoxide | C_5_H_11_NO_3_S | 0.2±0.1 | 0.1±0.0 |
|  |  | Ornithine | C_5_H_12_N_2_O_2_ | 0.9±0.2 | 0.2±0.1 |
|  |  | Ornithine-1,5-lactam | C_5_H_10_N_2_O | 0.1±0.0 | 0.0±0.0 |
|  |  | Proline | C_5_H_9_NO_2_ | 3.5±1.5 | 0.5±0.1 |
|  |  | *trans*-4-Hydroxy-proline | C_5_H_9_NO_3_ | 0.3±0.1 | 0.1±0.0 |
|  |  | Putrescine | C_4_H_12_N_2_ | 1.8±0.4 | 0.2±0.0 |
|  |  | Pyroglutamate | C_5_H_7_NO_3_ | 163±24.1 | 19.6±4.5 |
|  |  | Serine | C_3_H_7_NO_3_ | 42±11.6 | 1.4±0.7 |
|  |  | Threonine | C_4_H_9_NO_3_ | 9.3±2.3 | 0.6±0.2 |
|  |  | Tryptophan | C_11_H_12_N_2_O_2_ | 2.1±1.9 | 0.5±0.2 |
|  |  | Tyrosine | C_9_H_11_NO_3_ | 11.5±4.4 | 1.3±0.4 |
|  |  | Valine | C_5_H_11_NO_2_ | 14.4±4.1 | 1.5±0.3 |
|  |  | Phenylalanine | C_9_H_11_NO_2_ | 3.3±0.7 | 0.4±0.1 |
|  |  | 3-Cyano-alanine | C_4_H_6_N_2_O_2_ | 0.2±0.1 | 0.0±0.0 |
|  |  | *β*-Alanine | C_3_H_7_NO_2_ | 0.5±0.1 | 0.1±0.0 |
| **Sugars and sugar alcohols** | | | | | |
|  | - Polar metabolites in MeOH/H_2_O phase  - GC-TOF-MS | Fructose 6-P | C_6_H_13_O_9_P | 0.6±0.0 | 0.1±0.0 |
|  |  | Fucose | C_6_H_12_O_5_ | 4.7±0.9 | 3.6±0.8 |
|  |  | Galactinol | C_12_H_22_O_11_ | 0.3±0.1 | 0.4±0.1 |
|  |  | Galactono-1,4-lactone | C_6_H_10_O_6_ | 3.7±1.7 | 3.8±1.4 |
|  |  | Glycerol | C_3_H_8_O_3_ | 1.3±0.3 | 1.9±0.2 |
|  |  | Myo-inositol | C_6_H_12_O_6_ | 24.2±4.5 | 22.0±3.7 |
|  |  | Melibiose | C_12_H_22_O_11_ | 0.2±0.1 | 0.4±0.1 |
|  |  | Raffinose | C_18_H_32_O_16_ | 7.2±9.8 | 19.2±11.2 |
|  |  | Rhamnose | C_6_H_12_O_5_ | 5.4±1.1 | 2.1±0.4 |
|  |  | Threose | C_4_H_8_O_4_ | 0.1±0.0 | 0.9±0.2 |
|  |  | Xylitol | C_5_H_12_O_5_ | 1.0±0.2 | 0.8±0.2 |
|  |  | Sucrose | C_12_H_22_O_11_ | 65.1±8.0 | 65.1±8.5 |
| **Organic acids** | | | | | |
|  | - Polar metabolites in MeOH/H_2_O phase    - UPLC-C_18_-FT-MS/MS | Citric acid | C_6_H_8_O_7_ | 87.0±16.6 | 69.0±13.5 |
|  | - Polar metabolites in MeOH/H_2_O phase  - GC-TOF-MS | Benzoic acid | C_7_H_6_O_2_ | 0.4±0.4 | 0.3±0.1 |
|  |  | 4-Hydroxy-benzoic acid | C_7_H_6_O_3_ | 0.6±0.1 | 0.5±0.1 |
|  |  | 4-Amino-butanoic acid | C_4_H_9_NO_2_ | 4.8±1.0 | 0.7±0.2 |
|  |  | 2-Amino-butyric acid | C_4_H_9_NO_2_ | 0.4±0.1 | 0.1±0.0 |
|  |  | 2-Hydroxy-butyric acid | C_4_H_8_O_3_ | 0.1±0.0 | 0.1±0.0 |
|  |  | Fumaric acid | C_4_H_4_O_4_ | 0.9±0.2 | 4.8±0.9 |
|  |  | Glyceric acid | C_3_H_6_O_4_ | 1.3±0.3 | 2.9±0.4 |
|  |  | Gluconic acid | C_6_H_12_O_7_ | 3.2±1.0 | 1.6±0.4 |
|  |  | Glycolic acid | C_2_H_4_O_3_ | 0.1±0.0 | 0.9±0.1 |
|  |  | Pipecolic acid | C_6_H_11_NO_2_ | 0.8±0.3 | 0.3±0.1 |
|  |  | Pyruvic acid | C_3_H_4_O_3_ | 0.3±0.1 | 1.5±0.2 |
|  |  | Succinic acid | C_4_H_6_O_4_ | 0.3±0.0 | 0.5±0.1 |
|  |  | 1-Dehydro-ascorbic acid | C_6_H_6_O_6_ | 2.2±0.7 | 0.4±0.1 |
|  |  | Nicotinic acid | C₆H₅NO₂ | 1.0±0.3 | 1.2±0.3 |
| **Miscellaneous/Others** | | | | | |
|  | - Non -polar metabolites in MTBE phase  - UPLC-C_8_-FT-MS/MS | Di-methylenedioxy cinnamoylmethane | C_21_H_16_O_6_ | 93.2±9.4 | 62.6±5.4 |
|  |  | Sabadelin | C_35_H_62_O_3_ | 1.0±0.0 | 0.9±0.0 |
|  |  | Calebin A | C_21_H_20_O_7_ | 34.6±1.9 | 25.1±1.7 |
|  | - Polar metabolites in MeOH/H_2_O phase  - GC-TOF-MS | Nicotinamide | C_6_H_6_N_2_O | 0.01±0.0 | 0.01±0.0 |
|  |  | 2-Hydroxy-pyridine | C_5_H_5_NO | 0.8±0.1 | 0.9±0.1 |
|  |  | Riboflavin | C₁₇H₂₀N₄O₆ | 13.3±4.8 | 16.0±9.1 |
|  |  | Uracil | C_4_H_4_N_2_O_2_ | 0.04±0.0 | 0.1±0.0 |
|  |  | Urea | CH_4_N_2_O | 0.5±0.2 | 0.6±0.3 |
|  |  | Spermidine | C_7_H_19_N_3_ | 1.6±0.5 | 1.4±0.4 |
|  |  | Orthophosphoric acid | H₃PO₄ | 59.6±8.8 | 74.1±6.4 |
|  | - Polar metabolites in MeOH/H_2_O phase    - UPLC-C_18_-FT-MS/MS | Corchoionoside B (fatty acyl glycosides of mono- and disaccharides) | C_19_H_28_O_9_ | 69.6±7.3 | 70.2±4.7 |
|  |  | Drovomifoliol-*O*-glucopyranoside | C_20_H_32_O_10_ | 9.2±0.6 | 9.4±0.5 |
|  |  | Gibberellic acid (Growth regulator) | C_19_H_22_O_6_ | ND | 1.1±0.2 |
|  |  | Oleuropeoylsucrose (Saccharolipids) | C_22_H_36_O_13_ | 0.4±0.0 | 0.4±0.1 |
|  | - Non -polar metabolites in MTBE phase  - UPLC-C_8_-FT-MS/MS | *β*-Citraurin | C_30_H_40_O_2_ | 33.0±2.3 | 44.4±4.4 |
|  |  | *β*-Citraurol | C_30_H_42_O_2_ | 81.1±4.6 | 77.9±4.5 |

ND: not detected
